# Selective Separation of Highly Similar Proteins on Ionic Liquid-Loaded Mesoporous TiO_2_

**DOI:** 10.1021/acs.langmuir.1c03277

**Published:** 2022-03-06

**Authors:** Yihui Dong, Aatto Laaksonen, Mian Gong, Rong An, Xiaoyan Ji

**Affiliations:** † Department of Molecular Chemistry and Materials Science, Weizmann Institute of Science, Rehovot 76100, Israel; ‡ Energy Engineering, Division of Energy Science, Luleå University of Technology, Luleå 97187, Sweden; § Herbert Gleiter Institute of Nanoscience, Department of Materials Science and Engineering, Nanjing University of Science and Technology, Nanjing 210094, P.R. China; ∥ Department of Materials and Environmental Chemistry, Arrhenius Laboratory, Stockholm University, Stockholm SE-10691, Sweden; ⊥ Center of Advanced Research in Bionanoconjugates and Biopolymers, “Petru Poni” Institute of Macromolecular Chemistry, Iasi 700469, Romania; # State Key Laboratory of Materials-Oriented and Chemical Engineering, Nanjing Tech University, Nanjing 211816, China

## Abstract

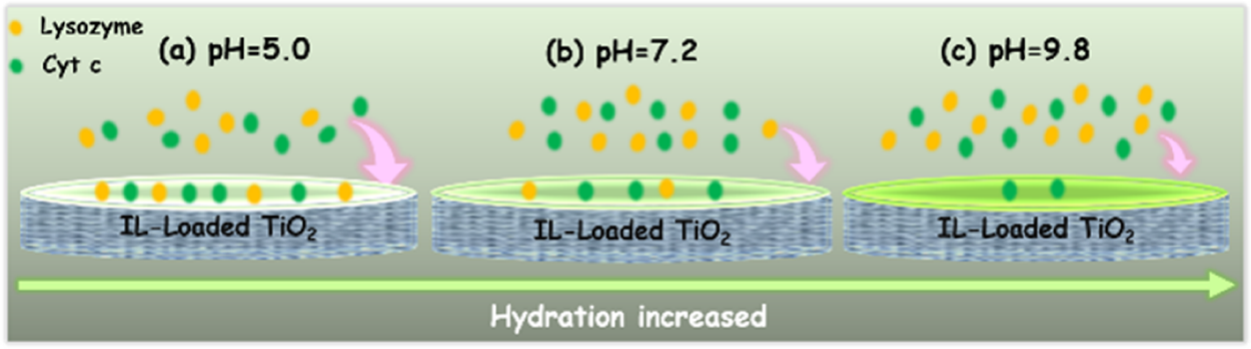

Separating proteins from their mixtures is an important process in a great variety of applications, but it faces difficult challenges as soon as the proteins are simultaneously of similar sizes and carry comparable net charges. To develop both efficient and sustainable strategies for the selective separation of similar proteins and to understand the underlying molecular mechanisms to enable the separation are crucial. In this work, we propose a novel strategy where the cholinium-based amino acid [Cho][Pro] ionic liquid (IL) is used as the trace additive and loaded physically on a mesoporous TiO_2_ surface for separating two similar proteins (lysozyme and cytochrome *c*). The observed selective adsorption behavior is explained by the hydration properties of the [Cho][Pro] loaded on the TiO_2_ surface and their partially dissociated ions under different pH conditions. As the pH is increased from 5.0 to 9.8, the degree of hydration of IL ions also increases, gradually weakening the interaction strength of the proteins with the substrates, more for lysozymes, leading to their effective separation. These findings were further used to guide the detection of the retention behavior of a binary mixture of proteins in high-performance liquid chromatography, where the introduction of ILs did effectively separate the two similar proteins. Our results should further stimulate the use of ILs in the separation of proteins with a high degree of mutual similarity.

## 1 Introduction

Great progress has been made in recent years in engineering biosurfaces for the chromatographic separation of proteins.^1^ The performance of separating proteins from mixtures relies predominantly on the regulation of protein interactions with substrates. These interactions can be tuned at nano/microscale surfaces by varying the surface properties of the substrates (i.e., size-^2−4^ and charge^2,5^-based separation) and the external environment (i.e., pH^6^ and ionic strength^7^). However, in previously published works, only the proteins with either comparable size or similar net charge could be separated. Whereas, the separation of highly similar proteins, that is, those with both similar size and charge [i.e., close isoelectric point (pI)], remains a challenge. To the best of our knowledge, no method is currently available to separate proteins with both similar size and charge.

Selective adsorption is the basic working principle in chromatography. However, in chromatographic separation, recyclability and regeneration of the substrate materials are important, but the complex chemical modifications of the substrates can easily lead to poor recyclability. It has been observed that for size- and charge-similar proteins, their molecular level details, in terms of residues or ligands, can still be quite different,^8,9^ resulting in different adsorptions. Distinct microscopic moieties of a protein can become more important factors, compared to the overall structure of the protein itself, in the characterization of the interaction of the protein with the substrate. Therefore, in regulating the interaction forces of proteins with substrates (i.e., their solid surfaces), variation of the surrounding microenvironment or modification of the chemical characteristics of the substrates can become decisive. The former option can often be a better alternative to preserve the desired substrate properties, in particular, the biocompatibility of certain materials such as TiO_2_-based chromatographic substrates.^7^

Because of their unique properties, including excellent chemical/thermal stability and tunable chemical structures, ionic liquids (ILs) have been introduced to several fields of biochemistry. This is very much because ILs can change the microenvironment with their highly heterogeneous microcompositions.^10−14^ Besides using ILs as solvents or media in bulk systems, they can also be used as additives in preparing IL-functionalized or chemically modified substrates. This has also been largely explored in biological fields.^15,16^ As stressed above, changing the microenvironment is a good alternative strategy to regulate the protein–substrate interactions, and loading ILs on the substrates can be an efficient way to achieve efficient recyclability and regeneration of the substrate materials. However, to the best of our knowledge, the underlying mechanisms, together with how the loading of ILs on the substrate changes the protein–substrate interactions both qualitatively and quantitatively in achieving the separation of size- and charge-similar proteins have not yet been investigated.

To describe the protein–substrate interaction forces quantitatively, a method has been developed to evaluate the interactions of proteins with biomaterial surfaces based on atomic force microscopy (AFM)^17−20^ by immobilizing the proteins on self-assembled monolayer (SAM)-functionalized AFM tip.^21^ This method provides quantitative information at the nanoscale by performing AFM adhesion measurements of protein interactions with substrates.^21−23^ We have previously used AFM to determine the molecular forces between the proteins and the TiO_2_ surface under different complex conditions, that is, surface roughness,^24^ pH conditions,^25^ heterogeneity,^26^ and ionic strength.^27^ These systematic measurements to determine quantitative interaction forces have shed light on understanding in more detail the underlying mechanisms from the microscopic perspective, serving as a guideline for the design of macroscopic experiments.

In the present work, loading ILs physically onto the substrates is carried out to change their microcomposition, and thereafter, in detail, the separation of similar proteins to clarify the underlying mechanisms. TiO_2_ is a favorable biomaterial for manipulating proteins due to its excellent biocompatibility and controllable structural properties, especially the long-term stability compared with other materials. Meanwhile, studies of the biointerface phenomena on biocompatible TiO_2_ are important for a variety of applications besides protein separation, such as medical devices, biosensors, drug delivery, and biodetection. Also, the geometric structures and the surface roughness of the mesoporous TiO_2_ used in this work can be varied directly by the simple control of the calcination temperatures of precursors,^24,28^ and these nano- and mesoporous materials, and their interactions with biomolecules, have been the research focus and stimulated studies on biomolecular adsorption and separation.^29,30^ A large number of ILs have been synthesized and characterized, among which the ILs with cholinium as the cation and amino acids as the anions are biocompatible, that is, bio-ILs, and appear to be promising solvents for selective extraction in bioprocesses.^31,32^ In this work, a commonly used bio-IL, choline proline ([Cho][Pro]),^33^ which is a counterpart of the bio-IL structure and is biodegradable and cheap, was loaded onto the mesoporous TiO_2_ as a composite substrate. Two proteins, lysozyme and Cyt *c*, with a similar globular size (∼1.5 nm in radius) and close pI values (∼10.0, see Figure S1), were used to form a binary mixture as the model separation system. The pH conditions were adjusted in the study to optimize the performance. Combining the selective adsorptions and the AFM-based adhesion force measurements, a systematic study was conducted, and the retention behavior of the proteins was detected with high-performance liquid chromatography (HPLC) for further verification of the separation performances.

## 2 Experimental Section

### 2.1 Materials

Cytochrome *c* [Cyt *c*, dimensions: 2.6 × 3.2 × 3.3 nm^3^, molecular weight (*M*_w_): 12.4 kDa] and lysozyme [dimensions: 3.0 × 3.0 × 4.5 nm^3^, *M*_w_: 14.4 kDa] were purchased from Bio Dee Bio-Tech Co., Ltd. (Beijing, China). 16-Mercaptohexadecanoic acid [HS(CH_2_)_15_COOH, 90%] purchased from Sigma-Aldrich Trading Co., Ltd., *N*,*N*-dimethyl formamide, triethylamine (99%), and trifluoroacetic anhydride (98%) purchased from J&K Scientific Ltd., and dichloromethane (99.5%) purchased from Sinopharm Chemical Reagent Co., Ltd., were used to functionalize AFM tips with proteins.

Three different pH conditions (5.0, 7.2, and 9.8) were chosen in the study, and the buffer with pH = 5.0 was prepared with 0.1 M acetic acid (CH_3_COOH, 99.5%, purchased from Shanghai Shenbo Chemical Co., Ltd.) and 0.1 M sodium acetate (CH_3_COONa, 99%, purchased from Shanghai Lingfeng Chemical Reagent Co., Ltd.) with a volumetric ratio of 3:7; that at pH = 7.2 was prepared with 0.1 M disodium hydrogen phosphate (Na_2_HPO_4_, 99%, purchased from Sinopharm Chemical Reagent Co., Ltd.) and 0.1 M potassium dihydrogen phosphate (KH_2_PO_4_, 99.5%, purchased from Shanghai Lingfeng Chemical Reagent Co., Ltd.) with a volumetric ratio of 6.7:3.3; and the one at pH = 9.8 was prepared with 0.1 M sodium carbonate (Na_2_CO_3_, 99.8%, purchased from Shanghai Lingfeng Chemical Reagent Co., Ltd.) and 0.1 M sodium bicarbonate (NaHCO_3_, 99.5%, purchased from Shanghai Lingfeng Chemical Reagent Co., Ltd.) with a volumetric ratio of 9:1. Deionized water was used in all the experiments.

### 2.2 Preparation of IL-Loaded TiO_2_

The preparation of the mesoporous TiO_2_ is based on our previous work,^24^ and these mesoporous TiO_2_ samples with different geometrical topographies were obtained at different calcination temperatures, that is, 300, 500, and 600 °C, which were named T300, T500, and T600, respectively. The biocompatible IL, choline proline ([Cho][Pro]), was synthesized according to the literature.^34,35^ The loading ratio is approximately 0.01 g-ILs/0.1 g-TiO_2_.^36^ In detail, 0.01 g of ILs ([Cho][Pro]) were dissolved in 60 mL of methanol, and then about 0.1 g of T300, T500, and T600 were, respectively, added. After stirring for 12 h, the IL-loaded TiO_2_ samples were placed in a rotary evaporator under a vacuum in a water bath at 60 °C to remove methanol. After that, the samples were put into a vacuum drying box at 60 °C for 24 h to ensure the methanol was removed thoroughly. The IL-loaded TiO_2_ samples were obtained and named IL-T300, IL-T500, and IL-T600, respectively.

### 2.3 Characterization

X-ray diffraction (XRD, Bruker D8, Cu Kα radiation) was used to measure the crystal phases of the samples. Fourier transform infrared (FT-IR) spectra were recorded using an FT-IR spectrophotometer (NEXUS 670). The N_2_ adsorption–desorption measurements (Micromeritics Tristar II 3020) were used to determine the structural properties. Thermogravimetric analysis (TGA, Model SDT 2960) was used to detect the weight loss of the samples.

### 2.4 Adsorption of the Binary Protein Mixture

About 10 mL of 0.01 M binary mixtures containing two different proteins (lysozyme and Cyt *c*) with 2 mg·mL^–1^ each were prepared with the buffers (pH = 5.0, 7.2, and 9.8), in which 2 mg·mL^–1^ is sufficient for the protein to reach maximum equilibrium adsorption.^24^ Each 200 mg mesoporous TiO_2_ sample (T300, T500, and T600 and IL-T300, IL-T500, and IL-T600) was mixed with the binary protein solutions at each pH value in the closed centrifuge tubes, and the samples in the centrifuge tubes were kept in the water bath at 30 °C and shaken at 180 rpm for 72 h to reach equilibrium. After that, the proteins were adsorbed on the TiO_2_ samples and separated by spinning the protein–TiO_2_ mixtures at 8000 rpm for 20 min. The supernatant concentration of proteins was determined by measuring the protein absorbance using an ultraviolet–visible (UV–vis) spectrophotometer, using an extinction coefficient of 38 940 cm^–1^ M^–1^ at λ = 280 nm for lysozyme and an extinction coefficient of 106 100 cm^–1^ M^–1^ at λ = 409 nm for Cyt *c*. Then, the amount of each protein bound to the samples was calculated from the difference between the initial and final concentrations of the protein in the solutions.

### 2.5 AFM Measurements

The measurements of the adhesion forces of each protein with TiO_2_ samples were performed using AFM (Dimension ICON, Bruker) in contact mode at room temperature. First, each protein molecule was separately immobilized on the SAM-functionalized gold-coated AFM tips (NPG-10, Si_3_N_4_, tip radius of 20 nm) by a chemical attachment following our previous work.^24^ In detail, first, AFM tips were immersed in the 1 mM HS(CH_2_)_15_COOH solution (50 vol % ethanol solvent) and maintained in an incubator for 12 h under dark conditions. Then, the tips were washed three times with ethanol and dried using nitrogen. Second, the tips were immersed in a mixture of trifluoroacetic anhydride (0.14 mL), triethylamine (0.28 mL), and *N*,*N*-dimethyl formamide (9.58 mL) for 20 min. Then, the tips were washed three times with dichloromethane and dried using nitrogen. During the last step, the tips were immersed in 5 mg·mL^–1^ of each protein solution separately at each pH and then washed with the corresponding buffer solutions three times and dried with nitrogen. The normal spring constant of the protein tip was calibrated to transform the normal load signals from volts (V) into the normal load (N). The adhesion forces were measured with the force–distance curve, and about 100 force–distance curves at the maximal adhesion force upon retraction were recorded at multiple randomly chosen spots and analyzed.

### 2.6 HPLC Retention Time Measurements

The retention behavior of the binary mixed proteins was investigated via HPLC (Agilent 1260 Infinity, USA) using TiO_2_ columns (the T500 sample was chosen as an example). The dimension × length of this column was 4.6 mm × 150 mm. The buffer solution was served as the mobile phase with a flow rate of 1 mL·min^–1^ at 25 °C. The 0.5 mg·mL^–1^ binary mixed protein solution in the buffer solution without ILs and in the one containing ILs (0.02 g of ILs/10 mL of protein solution), respectively, were separately injected (amount: 5 μL) into the column, and the adsorption profiles were monitored at 280 and 409 nm for lysozyme and Cyt *c*, respectively.

## 3 Results and Discussion

The present work was organized into six parts. In the first part, the characterizations of the mesoporous TiO_2_ after loading IL [Cho][Pro], and the thickness of the IL on the substrates were carried out. The adsorption selectivities of binary mixtures of lysozyme and Cyt *c* on the mesoporous TiO_2_ and the corresponding IL-TiO_2_ under three different pH conditions (5.0, 7.2, and 9.8) were conducted in the second part. In the third part, AFM-measured adhesion forces were used as support to rationalize and explain the different adsorption behaviors of two proteins. The mechanism behind the adsorption selectivity was discussed in the fourth part. In the fifth part, the HPLC measurements were used to determine the retention behavior of the binary mixture of lysozyme and Cyt *c* and to verify the role of ILs in protein separation. In the last part, the future perspectives were summarized to improve, optimize, and extend these systematic research studies.

### 3.1 Synthesis and Characterization

**Figure 1:**
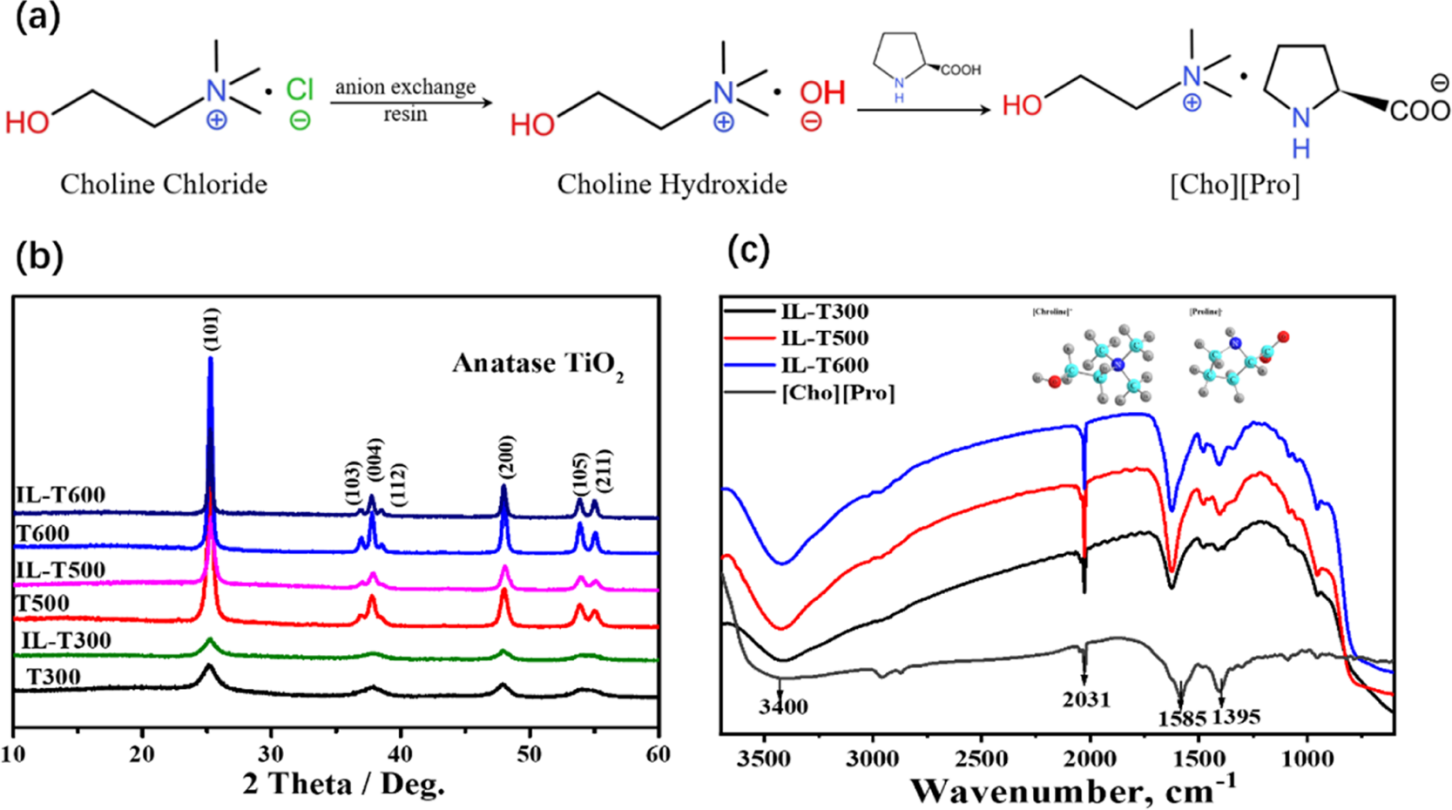
(a) Synthetic route of [Cho][Pro]; (b) XRD patterns of mesoporous TiO_2_ and the corresponding IL-loaded mesoporous TiO_2_ samples; and (c) FT-IR spectrum of [Cho][Pro] and IL-loaded mesoporous TiO_2_ samples. Inset: chemical structure of [Cho][Pro].

Figure 1a shows the synthetic route of [Cho][Pro], in which choline chloride was converted to choline hydroxide first, and then neutralized with proline to obtain [Cho][Pro].^35^ According to the chemical structure, [Cho][Pro] was one kind of cholinium-based amino acid IL, where both anionic and cationic counterparts are derived from natural sources and show excellent water solubility, showing good hydration properties after dissociation.^37^Figure 1b shows the XRD patterns of these mesoporous TiO_2_ samples with different geometric structures (T300, T500, and T600) and the corresponding IL-loaded ones (IL-T300, IL-T500, and IL-T600). The mesoporous TiO_2_ showed well-resolved diffraction peaks corresponding to the reflections of the anatase TiO_2_ materials, and the crystallinity increased when the temperature was raised. Meanwhile, the diffraction patterns of these mesoporous TiO_2_ samples were not changed after loading IL [Cho][Pro], which indicates that the structure of these mesoporous TiO_2_ samples was kept without any variation. The intensity of the diffraction patterns of IL-TiO_2_ was slightly changed, and this might be caused by the effect of [Cho][Pro] loading, which is consistent with the previous findings in the literature.^36^ The FT-IR spectrum was used to verify the existence of IL loaded on the TiO_2_ surfaces, as shown in Figure 1c. When comparing the FT-IR spectra with the pure [Cho][Pro], the peak at 3400 cm^–1^ represents the stretching vibrations of O–H and N–H, while the peaks at 1585 and 1395 cm^–1^ represent the stretching and torsional vibration of the COO– group, respectively. The peak at 2031 cm^–1^ represents the symmetric and asymmetric stretching vibrations of C–H, respectively. Both the XRD and FT-IR results demonstrate that the IL loading on these mesoporous TiO_2_ samples is indeed successful.

**Figure 2:**
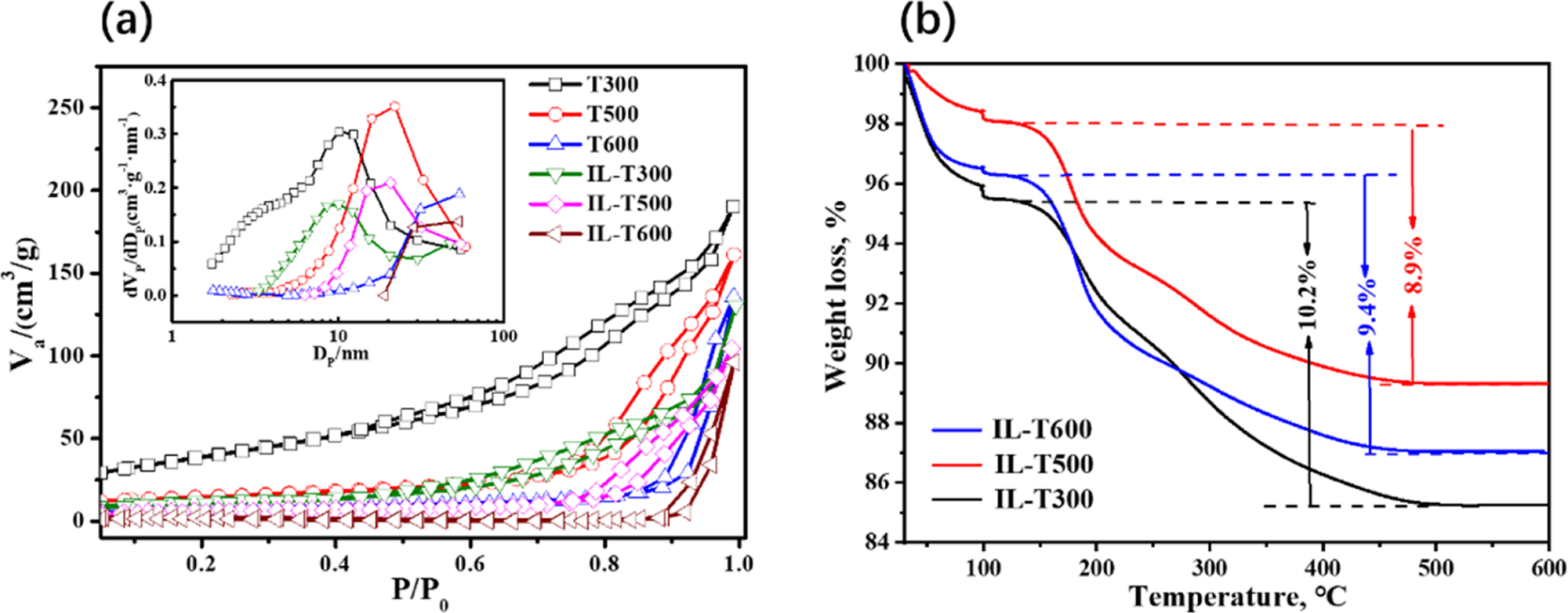
(a) N_2_ adsorption–desorption isotherms and pore size distribution curves of sintered mesoporous TiO_2_ and corresponding IL-TiO_2_ samples and (b) TGA experiment of the [Cho][Pro]-loaded TiO_2_ sample.

The N_2_ adsorption–desorption isotherm is displayed in Figure 2a, for both the mesoporous TiO_2_ and the corresponding IL-TiO_2_ samples, showing a typical isotherm of type IV and indicating the presence of well-developed mesopores in the samples. The mesopore structures of these samples were also displayed by scanning electron microscopy (SEM), showing that the mesopores had become larger with increasing the sintering temperature (see Figure S2). According to the structural parameters measured by Brunauer–Emmett–Teller (BET), listed in Table 1, the specific surface area and pore volume decreased, while the average pore size increased with the increase of the calcination temperatures, which is due to the higher temperatures resulting in the collapse of the pores. Therefore, both the crystallinity and the BET and porosity are related to the calcination temperature, where the mesoporous T300 prepared at the lowest calcination temperature (300 °C) has the lowest crystallinity and the highest BET and porosity.^28^ However, after loading ILs, the specific surface area and pore volume decreased due to the “entrance” of ILs into the pores, most likely bridging the small pores to form larger ones and leading to the increased average pore size. The TGA measurements were used to determine the content of IL [Cho][Pro] loaded on the mesoporous TiO_2_ surface, as shown in Figure 2b. The weight loss at the first step at 100 °C represents the free water loss on the TiO_2_ surface, and that at the second step ranging from 100 to 600 °C represents the loss of [Cho][Pro] with the approximate reductions (i.e., the loading amounts of [Cho][Pro]) of 10.2, 8.9, and 9.4 wt % for IL-T300, IL-T500, and IL-T600, respectively, which is almost identical to the approximate values of 10 wt % (0.01 g-ILs/0.1 g-TiO_2_). The different weight reduction indicates that the effective amount of the IL loaded on TiO_2_ samples differs in absolute terms, which is due to the different specific surface areas of the TiO_2_ samples. Furthermore, based on the TGA measurements of the effective surface area of TiO_2_ after immobilization and the density of ILs, the thickness of ILs on these TiO_2_ surfaces was calculated with the approximate values of 2.3, 4.5, and 15.1 nm for IL-T300, IL-T500, and IL-T600, respectively.

**Table 1:** BET Measured Structural Parameters of Mesoporous TiO_2_ and the IL-Loaded TiO_2_ Samples

sample	pore size, nm	BET surface area, m^2^·g^–1^	pore volume, cm^3^·g^–1^
T300/IL-T300	8.4/15.5	137.5/40.3	0.293/0.154
T500/IL-T500	19.5/22.2	50.3/17.6	0.249/0.148
T600/IL-T600	32.7/41.3	25.2/5.5	0.210/0.129

### 3.2 Selective Adsorption of Binary Mixtures

**Figure 3:**
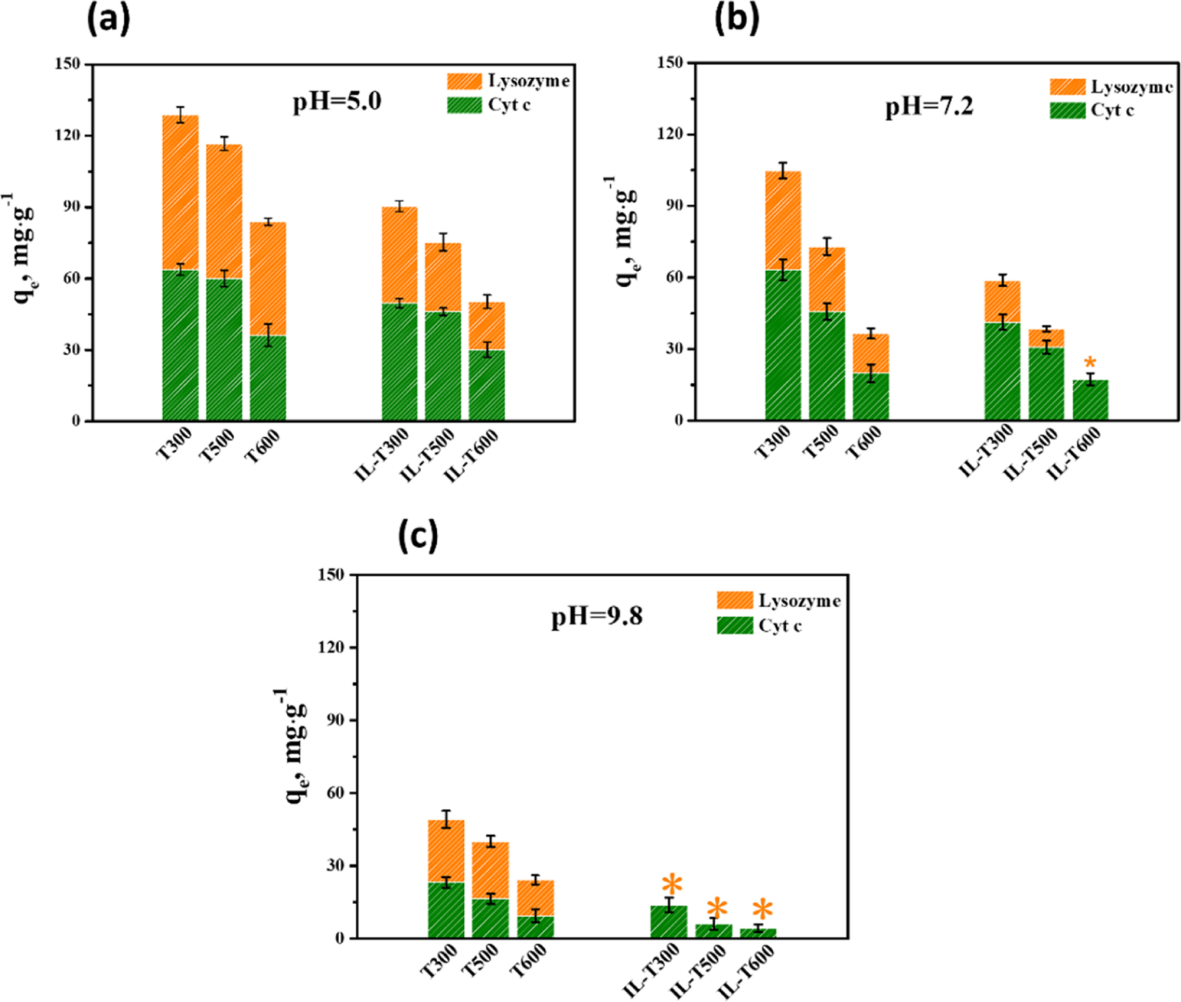
Binary mixed proteins’ adsorption capacities on mesoporous TiO_2_ (T300, T500, and T600) and corresponding IL-TiO_2_ (IL-T300, IL-T500, and IL-T600) at (a) pH = 5.0; (b) pH = 7.2; and (c) pH = 9.8.

Before quantitatively determining the selective adsorption, the UV–vis absorption spectra of each protein and the mixed proteins interacting with TiO_2_ and IL-TiO_2_ were studied, which verified the protein structural stability during the adsorption experiments (see Figure S3). The selective adsorption was then conducted systematically. The bar graphs in Figure 3 show the resulting adsorption capacity from a binary mixture of proteins (Cyt *c* and lysozyme) on both mesoporous TiO_2_ and IL-loaded TiO_2_ samples under different pH conditions. The overall height of the bar denotes the total amount of proteins bound to the TiO_2_ sample, while the dashed segments in different colors represent the shares that can be attributed to the different proteins involved. Interestingly, both proteins are attributed to roughly one-half of the total adsorption capacity of these mesoporous TiO_2_ samples with different surface areas under three different pH conditions, even though the total adsorption capacity decreased as the pH values increased. This indicates poor selective adsorption of these mesoporous TiO_2_ to separate similar proteins. However, there is a significant difference in the adsorption of these two proteins on the IL-loaded TiO_2_ samples, where lysozyme accounts for lower adsorption capacity than Cyt *c*, especially under pH = 9.8. This difference does suggest that the IL-TiO_2_ samples have a significant advantage in the selectivity of similar proteins.

After loading ILs, the total adsorption capacity was reduced. This can be explained because the ILs are partly dissolved and dissociated into cations and anions after adding them into the solution with water. Even though they were loaded on the TiO_2_ surface, the hydrated cations and anions did change the microenvironment in the solution. Meanwhile, these hydrated ions could form a strong hydration layer on the IL-loaded TiO_2_ surface, leading to a decrease in the molecular interaction forces and the total adsorption capacity of proteins.^38^

The formation of the hydration layer takes place on all the IL-TiO_2_ surfaces, while the hydration strength is different. Meanwhile, after hydration, a weak diffusion of the hydrated ions for the IL loaded on TiO_2_ is unavoidable. As the pH increases from 5.0 to 7.2, the diffusion of hydrated IL ions becomes weaker.^39^ Thus, the hydration strength of IL-TiO_2_ surfaces becomes stronger, leading to decreased total adsorption. Furthermore, based on the structure of IL [Cho][Pro], containing cations (N^+^) and anions (O^–^) together with one −OH, indicating that the diffusion of the hydrated ions for the IL loaded on TiO_2_ is weaker under the alkaline environment at pH = 9.8 than that at the acidic (pH = 5.0, Figure 3a) or neutral environment (pH = 7.2, Figure 3b). It implies that the hydration properties of IL-TiO_2_ could be the strongest at pH = 9.8, leading to a significantly decreased adsorption capacity (Figure 3c).

The striking selectivity of Cyt *c* over lysozyme at pH = 9.8, that is, there is almost no adsorption of lysozyme on IL-TiO_2_ (Figure 3c), could also be derived from the different properties (e.g., compositions and surface locations of residues) of these two proteins.^40,41^ This adsorption condition at pH = 9.8 is close to the pI of these two proteins, indicating that both proteins are close to neutral. Although the net charge of the protein vanishes at the pI, its surface still contains patches of positively and negatively charged amino acid residues.^42^ These charged patches of the protein drive the interactions on the charged surface and are related to the uniformity of charge distribution. The patches of positive and negative charges are distributed more uniformly on the lysozyme surface than on Cyt *c*, indicating the Cyt *c* molecules will be much easier to adsorb on the charged surfaces. Moreover, lysozyme is a rigid globular protein and is barely influenced by the surface polarity and solution environment due to its intrinsic structural stability.^43^ By contrast, Cyt *c* shows structural flexibility and is much more sensitive to external factors.^9^ It has also been reported that Cyt *c* is able to adsorb onto both negatively and positively charged surfaces and bare solid surfaces due to its variable orientation,^44^ resulting in higher adsorption than that of lysozyme.

Besides, the adsorption capacity also depends on the IL-loaded TiO_2_ samples, and those with a smaller surface area have a lower adsorption capacity. The different layer thickness of IL loading as evidenced by the TGA measurement could be the reason. Indeed, as the IL-TiO_2_ samples were added into the protein solution, the IL loaded on the TiO_2_ surface would be partially dissolved, dissociated, and hydrated in the solution until reaching equilibrium, that is, redistribution between the surface and the solution. The IL loadings on TiO_2_ decreased to about 7.6, 6.5, and 4.2 wt % for IL-T300, IL-T500, and IL-T600 (see Figure S4) after adsorption, reaching redistribution at equilibrium, and the thicknesses of ILs on these TiO_2_ surfaces were 1.7, 3.3, and 6.7 nm, respectively; that is, the IL layer is still thicker during the adsorption for IL-T600 compared to IL-T300 and IL-T500. Meanwhile, the different TiO_2_ samples have different surface/volume ratios, influencing the IL loading or coating. For example, the TiO_2_ with a large surface area possessed a thin layer thickness of the IL, and the change in the thickness is small after reaching redistribution at equilibrium, whereas the layer thickness of the IL on TiO_2_ with a small surface area was thicker and decreased relatively greatly due to the partial dissolution, dissociation, and hydration during the adsorption. Increasing the thickness of ILs could weaken the contribution of the substrate, enhance the hydration properties, and thus weaken the interaction between the adsorbed protein and the substrate.

In summary, for the TiO_2_ samples with IL-loading, the hydration not only decreases the adsorption capacity but also achieves remarkable selectivity. Thus, the IL-loaded TiO_2_ has great potential to enable selective adsorption of proteins with both similar size and charge. In particular, the IL-TiO_2_ samples demonstrate excellent protein separation performance at pH = 9.8, suggesting their application in chromatographic separation.

### 3.3 AFM-Based Adhesion Force Measurements

Gaining detailed knowledge of the binding behavior of proteins at liquid–solid interfaces provides valuable insight into bioseparations. To further understand the different adsorption behaviors of two similar proteins such as lysozyme and Cyt *c* on TiO_2_ and IL-TiO_2_ under different pH conditions, the adhesion forces between lysozyme/Cyt *c* molecules and the TiO_2_ (T500 and IL-T500 are chosen as examples) surfaces (*F*_n_ in nN) were determined separately. Before force detection, the AFM topographic image of the TiO_2_ surface was detected for those used for the adhesion force measurements, showing that the surface roughness increased slightly after loading ILs (see Figure S5). This implies the existence of the IL layer adsorbed on the TiO_2_ surface, which is consistent with the literature.^45^

**Figure 4:**
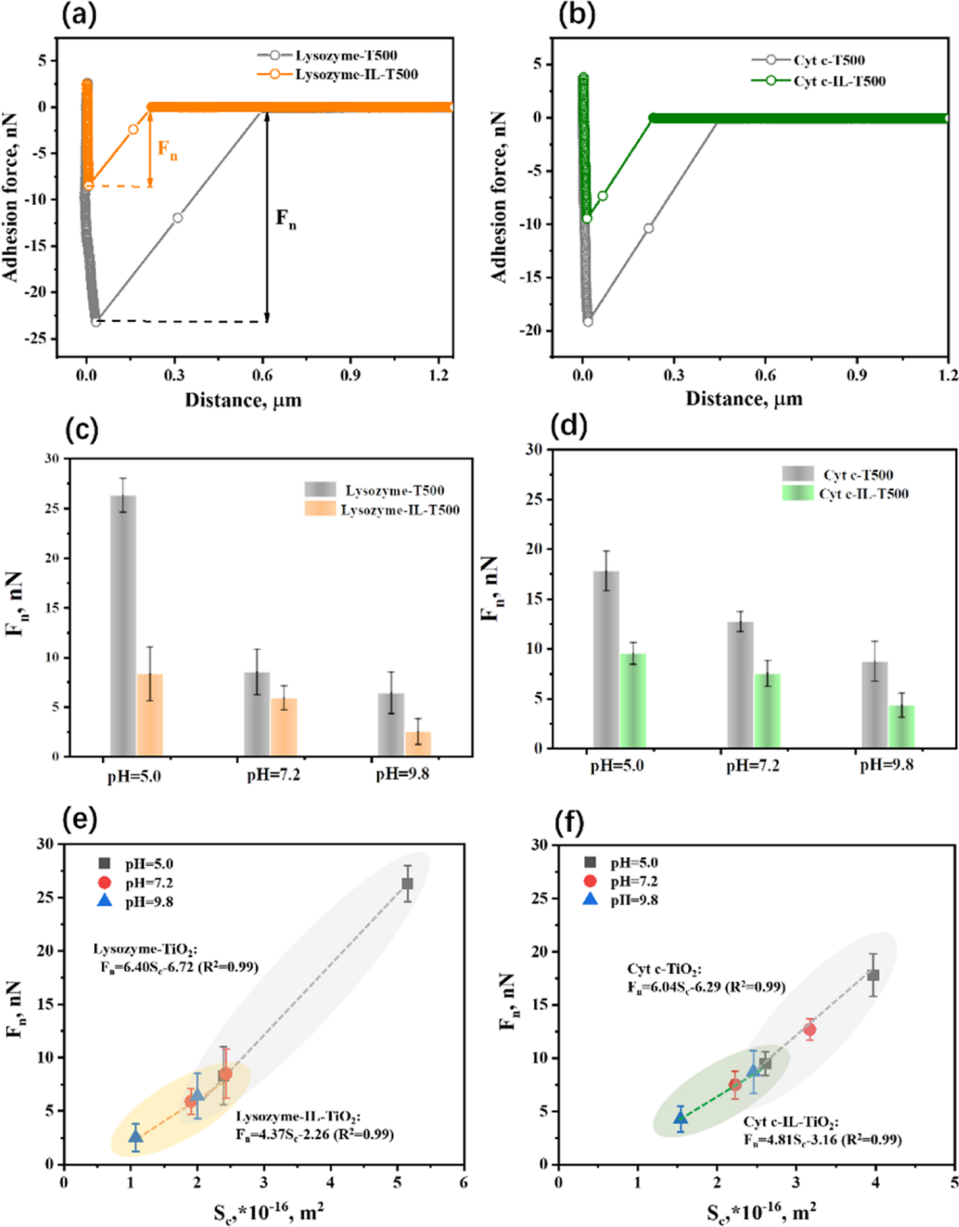
Typical force–distance curves for (a) lysozyme and (b) Cyt *c* on TiO_2_ (T500) and IL-TiO_2_ (IL-T500) at pH = 5.0; AFM-measured separate adhesion forces of (c) lysozyme and (d) Cyt *c* with mesoporous TiO_2_ (T500) and IL-TiO_2_ (IL-T500) under three different pH conditions; The adhesion force vs the effective contact area for (e) lysozyme and (f) Cyt *c* with T500 and IL-T500, respectively, under three different pH conditions. The dashed line is the guide to the eyes.

To detect the adhesion force, both the protein molecules were covalently attached to an AFM tip coated with gold to immobilize the proteins on the AFM tip. Because it is difficult to distinguish and decompose the adhesion force of each protein from the total adhesion force of mixed proteins, the adhesion force of proteins interacting with the solid surface was determined separately for lysozyme and Cyt *c*, instead of the binary mixed proteins attached to an AFM tip. The distributions of the adhesion forces are shown in Figures S6 and S7. Here, the adhesion force, *F*_n_, represented the total force between the protein clusters and substrates, corresponding to the maximum force jump during retraction. The typical force–distance curves shown in Figure 4a,b (pH = 5.0 as an example) corresponded to the lysozyme and Cyt *c* molecules interacting with TiO_2_ with and without ILs, respectively.

As shown in Figure 4c,d, the adhesion force of lysozyme with T500 at pH = 5.0 is larger than that of Cyt *c*. Furthermore, as the pH value increased from 5.0 to 7.2, the total adhesion force decreased, and the force of lysozyme was smaller than that of Cyt *c*. A stronger adhesion force for Cyt *c* was observed, compared with lysozyme at pH = 9.8. However, the adsorption capacities of lysozyme and Cyt *c* on mesoporous TiO_2_ were observed to be the same during the binary mixed selective adsorption. It is inconsistent with the adhesion force of each protein. This illustrates that both adsorption competition and interaction between two proteins could affect the final adsorption results.

After loading ILs, the adhesion forces for lysozyme and Cyt *c*, respectively, on IL-T500 decreased compared with those on T500 under three different pH conditions, which is also consistent with the trend over the adsorption capacity. Also, in the case of IL-T500, the adhesion force of lysozyme is lower than that of Cyt *c*, indicating a weaker interaction strength and providing a lower adsorption capacity, especially at pH = 9.8. The adhesion force in this part verified the different adsorption behaviors during the binary selective adsorption to some extent. After combining the adsorption and adhesion measurements, we could conclude that the addition of ILs, together with the pH conditions at 9.8, has advantages in separating these two proteins.

The adhesion force measured by AFM is related to the amount of the protein molecules adhered to the tip and the contact area between the protein and the surface, further resulting in a different interaction even with the same substrate. To discuss the adhesion force quantitatively, the effective contact area (*S*_c_ in m^2^) between the protein cluster-coated tip and the mesoporous TiO_2_ surface under three pH conditions was calculated with the Hertz and Johnson–Kendall–Roberts theories.^46^ The adhesion force of each protein on TiO_2_ with and without ILs per unit contact area (*F*_n_*/S*_c_ in nN/m^2^) was obtained according to the linear relationship in Figure 4e for lysozyme and Figure 4f for Cyt *c*, where the slopes were 6.40 and 4.37 for the lysozyme system, and 6.04 and 4.81 for the Cyt *c* system, without and with IL immobilization on TiO_2_, respectively. The effective contact area is obviously proportional to the total force, and the difference in these values of *F*_n_*/S*_c_ states the different numbers of the protein molecules interacting effectively with the substrates.

The molecular adhesion forces obtained in the present work are also consistent with the molecular forces quantitatively studied before from the aspect of molecular interaction at the nanoscale. For instance, the molecular force of lysozyme with a TiO_2_ surface is about 0.86 nN at pH = 7.2,^25^ and that for Cyt *c* with TiO_2_ is 1.32 nN,^47^ which is in accord with the findings in this work. Meanwhile, the molecular force of Cyt *c* on the IL-loaded TiO_2_ surface is weaker than that on TiO_2_ without ILs,^38^ which is consistent with the results obtained in this work.

### 3.4 Analysis of the Mechanism

Selective protein separation is critically important in a variety of bioapplications, and finding a way to illustrate the mechanism is of fundamental importance. Based on the discussion above, the detailed adsorption behavior sheds light on the different sensitivities of similar proteins to the substrates under specific conditions. The main mechanism is due to the hydration properties of ILs loaded on the mesoporous TiO_2_ surfaces and the induced electrostatic interactions. Furthermore, due to the inevitable diffusion of the ILs loaded on the TiO_2_ surface into the bulk, as discussed from the TGA results mentioned above, the zeta potential of IL [Cho][Pro] under different pH conditions was detected to inspect whether the IL will affect the chargeability of the system, influencing the selective adsorption. The results showed that the values of zeta potentials were close to zero (see Figure S8), indicating that the diffused IL ions are approaching full dissociation and completing their hydration rapidly. This suggests that the diffused IL ions do not alter the chargeability of the protein systems at different pH conditions.

**Figure 5:**
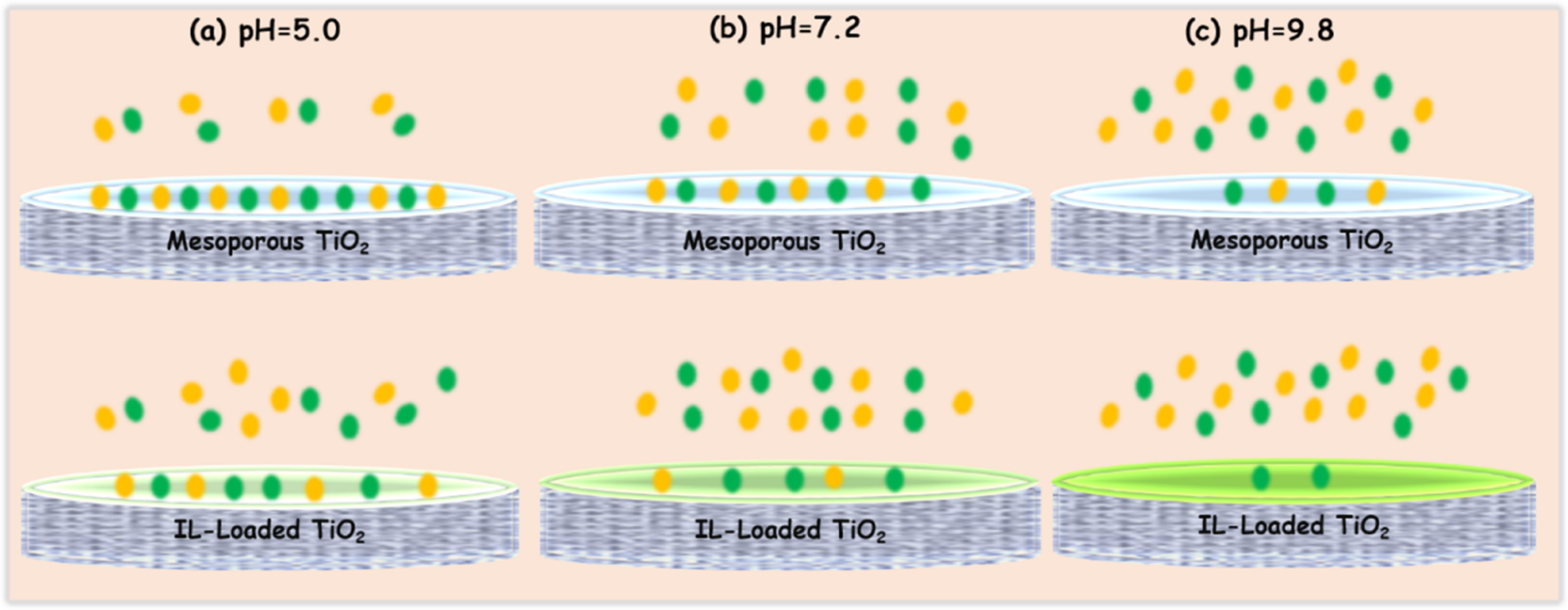
Schemes for the selective adsorption of binary mixed proteins on mesoporous TiO_2_ and on IL-TiO_2_ with hydration properties under different pH conditions: (a) pH = 5.0, (b) pH = 7.2, and (c) pH = 9.8.

The illustration in Figure 5 summarizes the mechanism of the distinct adsorption behavior of the binary mixed proteins and their interaction strength under three different pH conditions. As pH changed from 5.0 to 9.8, the total adsorption capacity of proteins on these mesoporous TiO_2_ samples decreased; however, poor selective adsorption of these mesoporous TiO_2_ was found when separating similar proteins. A striking difference was observed on these IL-loaded TiO_2_ samples. The hydration strength of IL loaded on the TiO_2_ surface at pH = 5.0 is the weakest, and the selective adsorption results of the two proteins are almost the same (Figure 5a). As the pH increased to 7.2, the total adsorption capacity decreased due to the increased hydration-induced decreased interaction strength (i.e., adhesion force), and the competitive adsorption performance of lysozyme was obviously reduced (Figure 5b). The hydration strength of IL was found to be strongest at pH = 9.8, leading to the decreased interaction strength (i.e., adhesion force) and the largely reduced total adsorption capacity of proteins, especially for the lysozyme (Figure 5c). Understanding these mechanisms is an important guide for applications, such as protein chromatography separation, studied in the following part.

### 3.5 HPLC Retention Behavior Measurements

**Figure 6:**
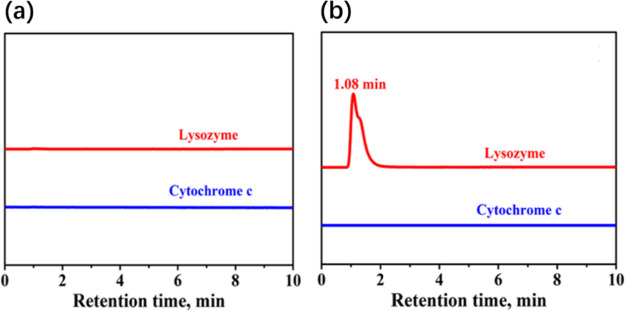
Retention behavior of binary mixed lysozyme and Cyt *c* passing through the (a) TiO_2_ column without the addition of ILs and (b) with ILs, determined by HPLC at pH = 9.8. The detection wavelength is 280 and 409 nm for lysozyme and Cyt *c*, respectively.

In principle, the performance of protein separation from mixtures by chromatography primarily relies on the regulation of different protein interactions with solid surfaces, where the retention behavior can represent the interaction strength between the protein and the solid surfaces. Also, the experimental HPLC process and the retention behavior of separated proteins relate to both the adsorption kinetics and capacity of mixed proteins. Unlike in our previous work, where we tested the retention behavior of the protein separately, in this work, the retention behavior was tested for the binary mixed proteins by passing through the TiO_2_ column (T500-column, laboratory homemade^25^) using HPLC. Based on the adsorption capacity and adhesion force measurements, the addition of ILs to mesoporous TiO_2_ together with the pH conditions at pH = 9.8 demonstrates the great potential for separating mixed proteins. Thus, the binary mixed proteins (lysozyme and Cyt *c*) passing through the TiO_2_ column with and without ILs were tested. The results are shown in Figure 6. Here, it needs to be emphasized that the IL is directly added into the binary mixed protein solution instead of loading the ILs onto the TiO_2_ column due to a certain degree of experimental difficulty.

Neither retention behavior nor a peak can be observed for binary mixed proteins passing through the TiO_2_ without ILs (Figure 6a), indicating stronger interactions between the mixed proteins and the TiO_2_ surface under this condition. After adding the IL, the retention behavior of lysozyme can be detected with a retention time of 1.08 min, whereas no signal was detected for Cyt *c* (Figure 6b). It indicated that the interaction strength of Cyt *c* with TiO_2_ is stronger than that of lysozyme after adding ILs. Our previous work did not show any retention behavior of lysozyme when passing through the TiO_2_ column using HPLC when the ionic strength is low.^48^ Furthermore, our recently published work enabled the retention behavior detection of lysozyme by adjusting the ionic strength.^7^ Different from our previous works, in the present work, we studied the addition of ILs into the biosystem to realize the binary mixed proteins separation, thereby stimulating a further use of ILs in protein separation and related applications in bioanalysis and environmental science. This HPLC retention behavior indicated that the ILs in the solution also have a significant effect on the protein separation, thereby verifying the feasibility and effectiveness of the proposed method by introducing ILs to the process of separating very similar proteins.

### 3.6 Future Perspectives

In this work, the introduction of ILs was used to achieve the separation of a pair of similar proteins as the first step, verifying the feasibility and effective selectivity. However, our preliminary experiments on a binary protein mixture still need to be further improved and optimized. For example, studying single-component adsorption is also essential to understanding the adsorption mechanism. As the method proposed in this work is effective in separating very similar proteins, systematic research from single to binary protein adsorptions and condition optimization will be conducted in the next step. Also, the loading ratio studied in this work is approximately 0.01 g-ILs/0.1 g-TiO_2_, and exploring the effects of loading ratios of IL and the supports on the protein separation behavior will be performed in the future. Furthermore, removing the IL in the separated samples is important to recover both the protein and ILs after the separation process. Inspired by the studies in the literature,^49^ dialysis measurement could be an efficient method to achieve this goal. In the future, exploring a suitable dialysis experiment and optimizing the conditions will be an important task to achieve both the recovery of protein and ILs after the separation process.^50^ Moreover, the experiments were also worth to extending achieve an effective separation for more complex protein systems, that is, ternary and quaternary systems.

## 4 Conclusions

In this work, IL [Cho][Pro] was used as the trace additive and loaded physically onto the mesoporous TiO_2_ surface, achieving the separation of binary mixed similar proteins (lysozyme and Cyt *c*) for the first time. A distinct difference in the competitive adsorption behavior was observed and explained based on the different hydration properties of the IL under three different pH conditions. As the pH increased from 5.0 to 9.8, the total adsorption capacity decreased, and the reduction of lysozyme was larger, both on the mesoporous TiO_2_ substrates with and without ILs. The hydration of IL ions weakened the interaction strength of proteins with the substrates, especially for the lysozyme during the competitive adsorption at pH = 9.8. The results of the adhesion force of each protein with the substrate detected with AFM are consistent with the observation of their adsorption behaviors. That is, the introduction of the IL weakened the interaction strength, especially for the lysozyme. These findings were further verified through the retention behavior tested by HPLC; that is, adding the IL can effectively separate the very similar proteins. Anyhow, our preliminary results should stimulate interest in using ILs in separating proteins with a high degree of similarity, and to exploit much more effective and optimized ILs in fields such as biodetection and bioanalysis, which are of great importance.

## Supporting Information Available

Zeta potentials titration curves of two proteins; SEM results of mesoporous TiO_2_; UV–vis spectra of proteins; TGA experiment of IL-loaded TiO_2_; AFM images of the TiO_2_ and IL-TiO_2_; distribution histograms of adhesion force of lysozyme on T500 and IL-T500 at different pH conditions; distribution histograms of adhesion force of Cyt *c* on T500 and IL-T500 at different pH conditions; and zeta potentials of IL [Cho][Pro] at three different pH conditions (PDF)
