# Supplementary material for: Selective Separation of Highly Similar Proteins on Ionic Liquid-Loaded Mesoporous TiO2
Source: Langmuir. 2022 Mar 6;38(10):3202–11. doi: 10.1021/acs.langmuir.1c03277 (PMC8928471; doi:10.1021/acs.langmuir.1c03277)
Supplement: Supplementary file 1 — la1c03277_si_001.pdf [file la1c03277_si_001.pdf]

Supporting information

Selective separation of highly similar proteins on ionic liquid-loaded mesoporous TiO_2

Yihui Dong[a], Aatto Laaksonen[b,d,e,f], Mian Gong[c], Rong An[c]*, and Xiaoyan Ji[b]*

[a]*Department of Molecular Chemistry and Materials Science, Weizmann Institute of Science, Rehovot, 76100, Israel.*

[b]*Energy Engineering, Division of Energy Science, Luleå University of Technology, 97187 Luleå, Sweden.*

[c]*Herbert Gleiter Institute of Nanoscience, Department of Materials Science and Engineering, Nanjing University of Science and Technology, Nanjing 210094, P.R. China.*

[d]*Department of Materials and Environmental Chemistry, Arrhenius Laboratory, Stockholm University, SE-10691 Stockholm, Sweden.*

[e]*Center of Advanced Research in Bionanoconjugates and Biopolymers, ''Petru Poni'' Institute of Macromolecular Chemistry, Iasi 700469, Romania.*

[f]*State Key Laboratory of Materials-Oriented and Chemical Engineering, Nanjing Tech University, Nanjing 211816, China.*

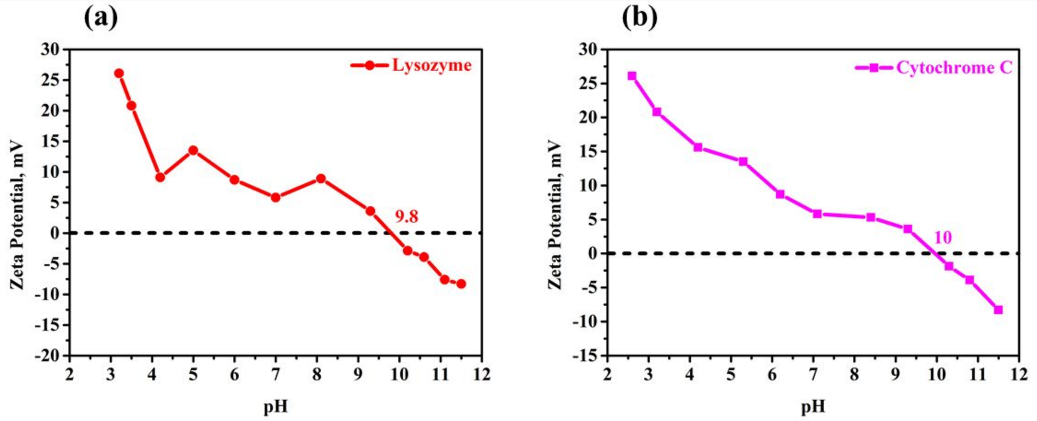

Figure S1. Zeta potentials titration curves of (a) lysozyme and (b) Cyt c.

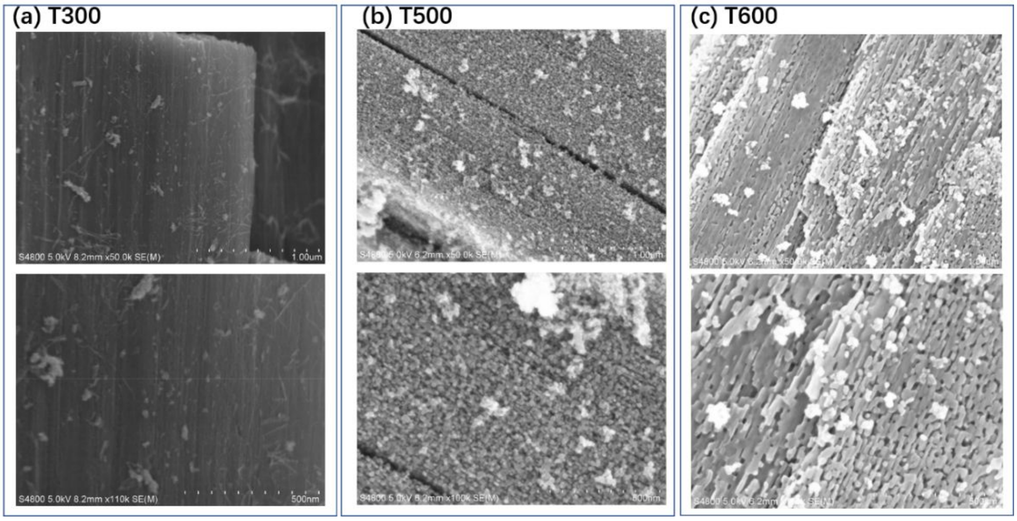

Figure S2. SEM results of mesoporous TiO_2: (a) T300 (bar: 1 um and 500 nm); (b) T500 (bar: 1 um and 500 nm); (c) T600 (bar: 1 um and 500 nm).

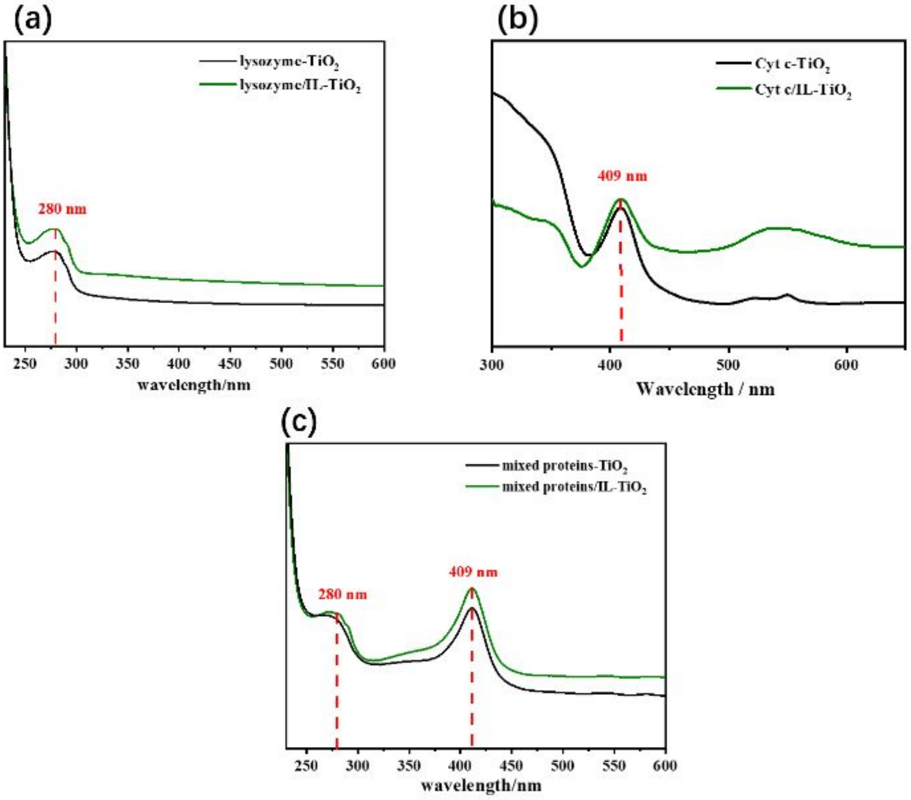

Figure S3. UV-vis spectra of (a) lysozyme, (b) Cyt c, and (c) mixed lysozyme and Cyt c proteins absorption on TiO_2 and IL-TiO_2, respectively.

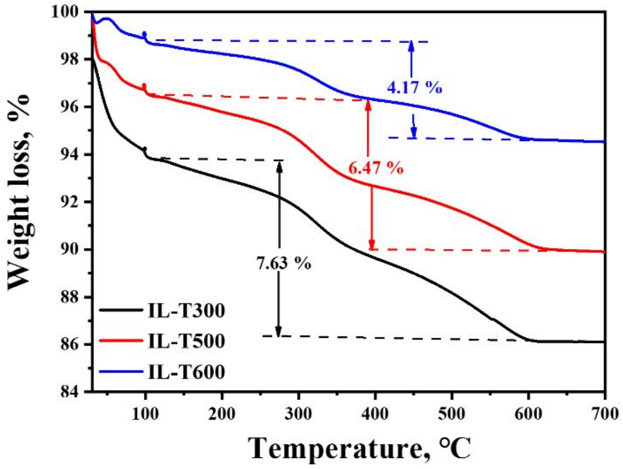

Figure S4. TGA experiments of [Cho][Pro]-loaded TiO_2 sample after the adsorption.

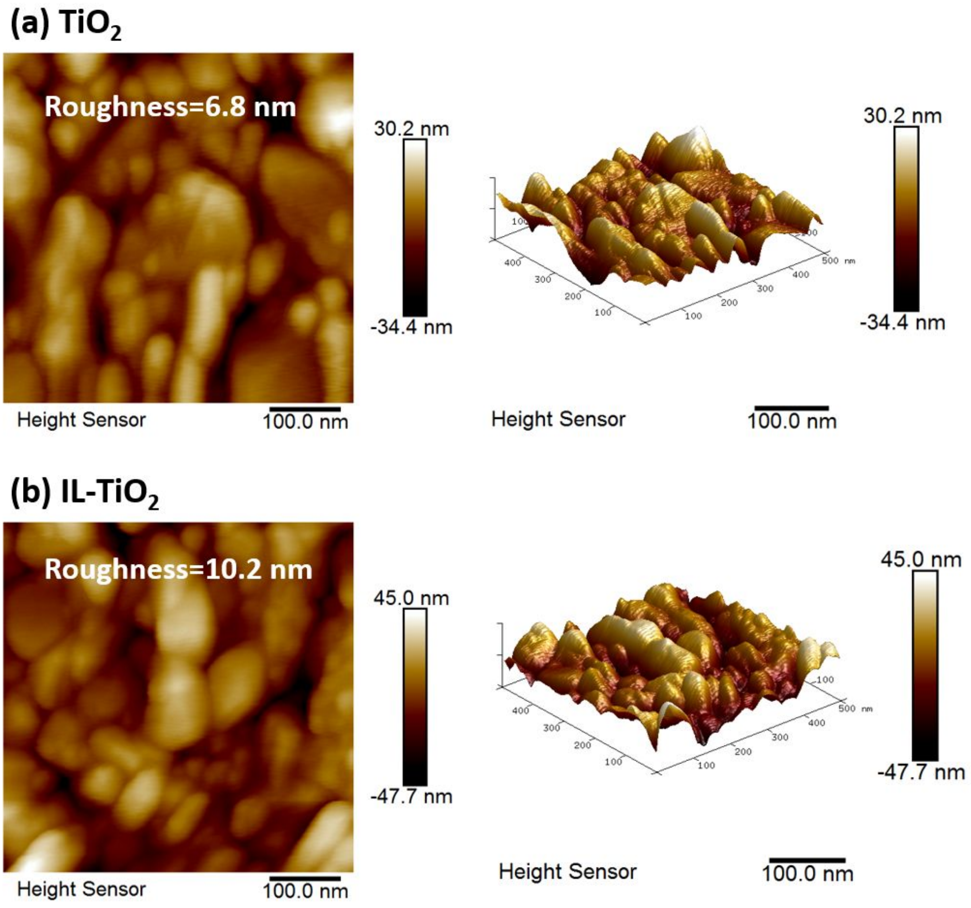

Figure S5. AFM topographic images (2 D and 3D) of the (a) TiO_2 surface and (b) IL-TiO_2 surface.

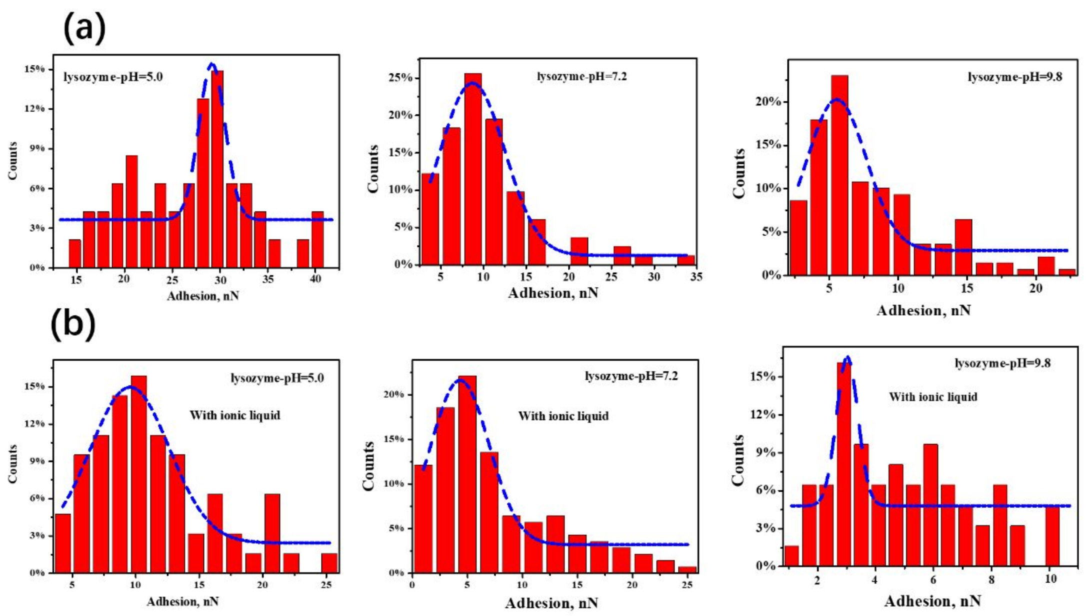

Figure S6. Distribution histograms of adhesion force of (a) lysozyme and T500; (b) lysozyme and IL-T500, at different pH conditions.

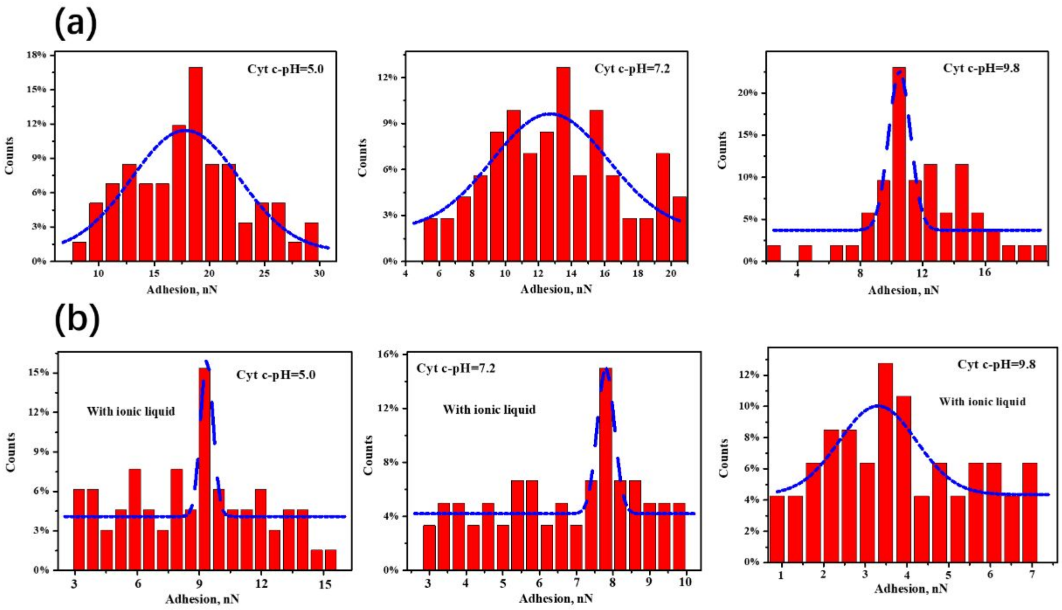

Figure S7. Distribution histograms of adhesion force of (a) Cyt c and T500; (b) Cyt c and IL-T500, at different pH conditions.

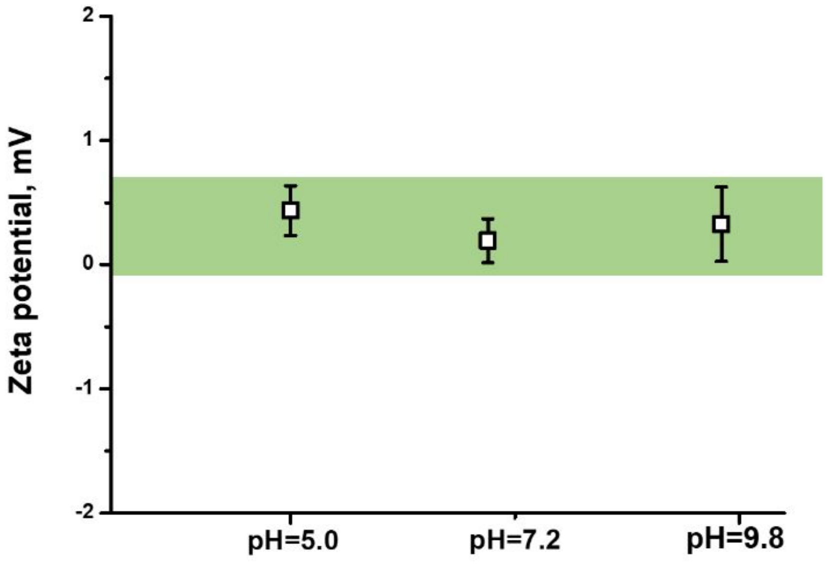

Figure S8. Zeta potentials of ILs [Cho][Pro] at three different pH conditions.
